# 3D-Printed Carbon-Based Conformal Electromagnetic Interference Shielding Module for Integrated Electronics

**DOI:** 10.1007/s40820-023-01317-w

**Published:** 2024-01-12

**Authors:** Shaohong Shi, Yuheng Jiang, Hao Ren, Siwen Deng, Jianping Sun, Fangchao Cheng, Jingjing Jing, Yinghong Chen

**Affiliations:** 1 State Key Laboratory of Featured Metal Materials and Life-Cycle Safety for Composite Structures, School of Resources, Environment and Materials, Guangxi University, No. 100, Daxuedong Road, Nanning, 530004 People’s Republic of China; 2 State Key Laboratory of Polymer Materials Engineering, Polymer Research Institute of Sichuan University, Sichuan University, No. 24 South Section 1, Yihuan Road, Chengdu, 610065 People’s Republic of China

**Keywords:** 3D printing, Carbon-based nanoparticles, Conformal electromagnetic interference shielding, Integrated electronics

## Abstract

Electromagnetic interference shielding (EMI SE) modules are the core component of modern electronics. However, the traditional metal-based SE modules always take up indispensable three-dimensional space inside electronics, posing a major obstacle to the integration of electronics. The innovation of integrating 3D-printed conformal shielding (c-SE) modules with packaging materials onto core electronics offers infinite possibilities to satisfy ideal SE function without occupying additional space. Herein, the 3D printable carbon-based inks with various proportions of graphene and carbon nanotube nanoparticles are well-formulated by manipulating their rheological peculiarity. Accordingly, the free-constructed architectures with arbitrarily-customized structure and multifunctionality are created via 3D printing. In particular, the SE performance of 3D-printed frame is up to 61.4 dB, simultaneously accompanied with an ultralight architecture of 0.076 g cm^−3^ and a superhigh specific shielding of 802.4 dB cm^3^ g^−1^. Moreover, as a proof-of-concept, the 3D-printed c-SE module is *in situ* integrated into core electronics, successfully replacing the traditional metal-based module to afford multiple functions for electromagnetic compatibility and thermal dissipation. Thus, this scientific innovation completely makes up the blank for assembling carbon-based c-SE modules and sheds a brilliant light on developing the next generation of high-performance shielding materials with arbitrarily-customized structure for integrated electronics.

**Supplementary Information:**

The online version contains supplementary material available at 10.1007/s40820-023-01317-w.

## Highlights

- 3D printable functional inks incorporated with graphene and carbon nanotube nanoparticles were well-formulated by manipulating their rheological performance
- The frame with ultralight structure (0.076 g cm^−3^) and high-efficiency electromagnetic interference shielding (61.4 dB) was assembled
- 3D-printed c-SE module was in situ integrated onto the electronics, affording multiple functions of electromagnetic compatibility and thermal dissipation.

**Supplementary Information:**

The online version contains supplementary material available at 10.1007/s40820-023-01317-w.

## Graphical abstract

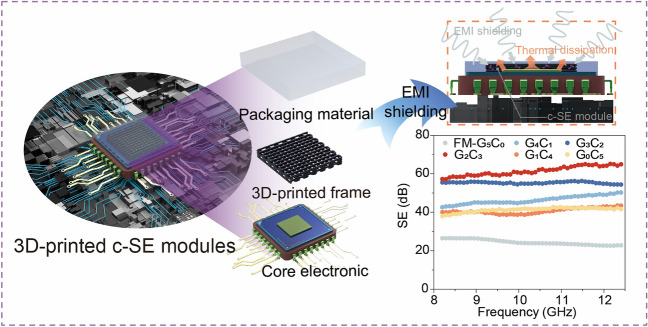

## Introduction

The Internet of Things (IoT) system has accelerated the rapid development of wireless communication networks, which relies on millimeter-level electromagnetic waves (EMWs) to achieve the information propagation and interaction of advanced electronics [1–3]. With the wide-spread popularity of 5G or future 6G wireless communication technology, EMWs have brought great convenience to national innovation and daily life, widely serving in smart sensing, navigational positioning, satellite communication, telemedicine, and other technological fields [4–7]. In this regard, the undesirable electromagnetic radiation generated by EMWs has attracted considerable attention because of its probable hazards for electronic security and human health [8–11]. The electromagnetic (EM) functional materials play a crucial role in solving EM interference problem [12–14]. Generally, the traditional approach to achieving electromagnetic compatibility involves the metal-based electromagnetic interference shielding (EMI SE) modules for attenuating EMWs energy [15–17]. Nevertheless, these SE modules would take up additional three-dimensional space inside electronics, which pose a major obstacle to the integration and miniaturization of electronics, and meanwhile, the existing air gap between elements may cause thermal management issue for the whole framework [18, 19]. In addition, the intrinsic deficiencies of metals, e.g., high density, difficulty to form, and potential environmental pollution also constrain their practical applications [20]. Hence, employing new ingredients and designing appropriate structures are the anticipated requirements for promoting the development of EMI SE materials in multi-scenario electromagnetic environments.

Advanced carbon nanomaterials, typically for carbon nanotube (CNT) [17, 21] and graphene (Gr) [22, 23], have been regarded as attractive candidates instead of metals owing to their prominent characteristics, e.g., robust electrical conductivity, well-fitting for EMI shielding. Currently, various strategies have been developed to fabricate carbon-based EMI materials by incorporating carbon nanoparticles into different supporting templates, e.g., ceramics, thermoplastic polymers, etc. [24, 25]. However, the poor interaction between carbon nanoparticles and matrix always limits the assembly and fabrication of multifunctional materials, especially in loading with high-concentration nanoparticles. Cellulose as a biomass polymer, mainly derived from plants, presents a promising potential in promoting the uniform dispersion of Gr and CNT nanoparticles in solution systems, since there exist abundant functional groups in molecular chains, imparting a positive contribution to the interaction between cellulose and carbon nanoparticles, thus realizing a strong interfacial binding in their composites [26, 27]. Hence, cellulose and its derivatives as organic templates were employed to develop some novel and versatile EMI shielding materials [28, 29].

Except for high-performance EMI materials, the optimization of shielding structures may expand their potential advantages in electromagnetic compatibility, better adapting to the miniaturization of integrated electronics [20, 30, 31]. Recently, a novel concept of “conformal-shielding” (c-SE) has been innovatively purposed, which means that the shielding layer is fully integrated with the packaging materials, thereby eliminating the need for additional space to complete SE functions [32, 33]. Noteworthily, the design principle of c-SE module lies in the arbitrary customization of structures according to electronics [34, 35]. In this regard, various techniques, including electroplating, spraying, and sputtering methods, are applied to achieve the c-SE module in integrated electronics [36–38]. However, these traditional methods are mainly serving for metal-based materials, there is almost no relevant technique for satisfying the manipulation of advanced carbon-based c-SE module to the best of our knowledge. Therefore, the innovation of manufacturing strategy suitable for carbon-based materials, has been attracted considerable attention from scientific vision [35, 39]. 3D printing technology, owing to the unique layer-by-layer manufacturing manner, illuminates infinite possibilities in designing and fabricating the novel architectures with arbitrarily-customized structures, thereby a serious of 3D printable carbon-based functional materials with free-constructed structures are reported [40–42]. Our group also concentrates on the development and utilization of 3D printable functional materials [43, 44]. However, employing 3D printing technology to manufacture carbon-based c-SE modules with ideal SE peculiarity has not been successfully achieved, because of the exiting challenges in accurately manipulating 3D printable carbon-based materials, and programmatically assembling target SE modules suitable for integrated electronics.

In this article, the well-formulated 3D printable functional inks, involving the elaborated manipulation of Gr@CNT nanoparticles with various hybrid proportions, were fabricated with the assistance of cellulose as adherent templates that delivered unique capabilities to resolving the interfacial issue between carbon nanoparticles and matrix, and affording desired rheological behaviors for direct ink writing (DIW) 3D printing. As expected, the 3D-printed patterns with arbitrarily-customized structures and ideal functionalities were manufactured, where the EMI SE efficiency of the optimal one was up to 61.4 dB, accompanied with 802.4 dB cm^3^ g^−1^, far beyond the previously reported SE materials [45–47]. More impressively, as a proof-of-concept, the 3D-printed c-SE modules were successfully designed and integrated with the packaging materials, which performed prominent SE function and thermal management capability for electronics. Overall, the scientific innovation created in this work paves a novel way for manufacturing carbon-based c-SE modules for integrated electronics, and unleashes a promising illumination for the next-generation SE materials with lighter, stronger, and fitter characteristics.

## Experimental Section

### Materials

Multi-walled carbon nanotubes (CNT, average length: ~ 1.5 μm, average diameter: ~ 9.5 nm) were provided by Nanocyl S.A., Belgium. Graphene (Gr, thickness range: 3.4 ~ 8.0 nm, flake size: 5 ~ 20 μm) was supplied by Suzhou Tanfeng Graphene Technology Co., Ltd., China. Carboxylated cellulose nanofibers (CNF, carboxyl content: 1.2 mmol g^−1^, chain length range: 1 ~ 3 μm, average diameter: ~ 7 nm) were purchased from Guilin Qihong Tech. Co., Ltd., China. Polyvinylpyrrolidone (PVP, average molecular weight: 101,200 ~ 110,000) was provided by Macklin Co. Ltd, China. Deionized water was supplied by Kelong Chemical Reagent Factory, China. All chemicals were used directly without purification treatment.

### Preparation of 3D Printable Gr@CNT Inks

1.0 g of Gr@CNT nanoparticles with a mass ratio of *x:y* (5:0, 4:1, 3:2, 2:3, 1:4, and 0:5), 0.3 g of CNF, and 0.15 g of PVP as a surfactant were dispersed in 300 mL deionized water under the ultrasonic treatment at 2 °C for 0.5 h by an ultrasonic cell disruptor (Ymnl-1800Y, Nanjing YMNL Instrument and Equipment, China). Noticeably, the detailed compositions of carbon-based functional inks are provided in Table S1. After uniform dispersion, the solution was mechanically stirred and concentrated at 80 °C to obtain the pseudo-solid inks (14.5 g). Then, the inks were centrifuged to remove the internal bubbles. After treatment, the prepared inks were stored at 4 °C for 3D printing. It is worth noting that the proportion of carbon-based inks were well-formulated by the principles of 3D printability and functionality. Particularly, the lacking of CNF would deteriorate the 3D printability of carbon-based ink, whereas the excessive CNF would sacrifice the electrical conductivity of as-fabricated frames, resulting in a weak EMI SE performance for materials. Moreover, for the sake of convenience, the Gr@CNT functional inks were signed as G_*x*_C_*y*_ to represent the relative ratio of Gr and CNT, where “*x*” and “*y*” represent the relative ratio of Gr and CNT in Gr@CNT nanoparticles, e.g., G_5_C_0_, G_4_C_1_, G_3_C_2_, G_2_C_3_, G_1_C_4_, and G_0_C_5_.

### Direct Ink Writing (DIW) 3D Printing of Gr@CNT Frames

According to the pre-programmed 3D printing program, the as-prepared Gr@CNT functional ink was loaded into the syringe and extruded by a desktop dispenser (TS-200BN, Shenzhen Tensun Precision Equipment, China) with a nozzle diameter of 800 μm, a step resolution of 20 μm, an appropriate pressure of 30 psi, and a writing speed of 5 mm s^−1^ (more detailed printing information is supported in Table S2). After printing, the assembled samples were placed into an ultra-low temperature freezer (MDF-382, Panasonic Appliances Cold Chain, Co. Ltd, China) and freeze for 2 h, and then moved to a freeze dryer (Ymnl-10N, Nanjing YMNL Instrument and Equipment Co. Ltd., China) for freeze-drying 10 h to obtain 3D-printed Gr@CNT frames.

### Characterization

The Zeta potentials of CNF, Gr@CNT and Gr@CNT/CNF dispersions with a dilutional concentration of ~ 5.0 mg mL^−1^ were measured by a Malvern Zetasizer NANO-ZS (Malvern Instruments, Worcestershire, UK). The rheological properties of Gr@CNT functional inks with a diameter of 25 mm and a gap of 1 mm were evaluated at 25 °C by a rotational rheometer (AR2000ex, TA Instruments, USA), the oscillatory angular frequency sweep was assessed in the range of 0.01–100 rad s^−1^ at a fixed strain of 5%, and the time sweep was tested at a step change of shear rates in 0.01 and 10 s^−1^. The oscillatory stress sweep was conducted on a shear rate range of 0.1–1000 Pa at a fixed angular frequency of 1 rad s^−1^. The thermal conductivity (*λ*) of packaging material and Gr@CNT frames with a size of 10 × 10 × 2.5 mm^3^ was tested by a laser flash analyzer (LFA467, NEXTZSCH, Germany), and the representative infrared images were obtained using an infrared camera (FLIRONE Pro, FLIR, USA). The surface and cross-sectional morphologies of 3D-printed frames were characterized by a field-emission scanning electron microscopy (SEM) (SU8020, Hitachi, Japan). The electrical conductivity of 3D-printed frames was evaluated by a multifunctional digital four-probe tester (ST2258C, Suzhou Crystal Electronic Co., Ltd., China). The radiation intensity generated by electronics of mobile phone was recorded by a radiation test instrument (620A, Ningbo Kemai Instrument and Equipment, China). The scattering parameters (S_11_, S_22_, S_12_ and S_21_) of 3D-printed frames with a diameter of 13.0 mm and a thickness of ~ 2.50 mm and compacted sample with a diameter of 13.0 mm and a thickness of ~ 0.40 mm in the 2.0–6.0 GHz and 8.2–12.4 GHz frequency range were measured by a vector network analyzer (N5230, Agilent Technologies, USA). The relevant electromagnetic characteristics of EMI shielding materials, e.g., absorption shielding (SE_A_), reflection shielding (SE_R_), total SE (SE_total_), specific SE value (SSE) and skin depth (*δ*) were calculated by the scattering parameters according to Eqs. (1–7) [31, 43, 48].

1 $$R = \left| {S_{11} } \right|^{2} = \left| {S_{22} } \right|^{2} ;\;T = \left| {S_{12} } \right|^{2} = \left| {S_{21} } \right|^{2}$$

2 $$A = 1 - R - T$$

3 $${\text{SE}}_{{\text{A}}} = - 10\log \left( {T/\left( {1 - R} \right)} \right)$$

4 $${\text{SE}}_{R} = - 10\log \left( {1 - R} \right)$$

5 $${\text{SE}}_{{{\text{total}}}} = {\text{SE}}_{{\text{R}}} + {\text{SE}}_{{\text{A}}} + {\text{SE}}_{{\text{m}}}$$

6 $${\text{SSE}} = {\text{SE}}_{{{\text{total}}}} /\rho^{\prime}$$

7 $$\delta= \sqrt {1/\pi f\sigma \mu }$$

where *R*, *T*, and *A*, represent reflection coefficient, transmission coefficient, and absorption coefficient, respectively; SE_A_, SE_R_, and SE_m_ represent absorption attenuation, reflection attenuation and multiple-internal reflection attenuation; SE_total_ and SSE represents the total EMI SE value and specific SE value; *ρ'* represents apparent density, according to the equation: *ρ'* = *m/V*, where *m* and *V* are the weight and the apparent volume of 3D printing models, respectively; besides, the *ρ'* of compacted sample is 0.692 g cm^−3^; *f* represents frequency; *σ* and *μ* represents electrical conductivity and permeability. Noticeably, when SE_total_ > 10 dB, SE_m_ could be neglected [43].

## Results and Discussion

### Rheological Performance and 3D Printability of Gr@CNT Functional Inks

**Fig. 1:**
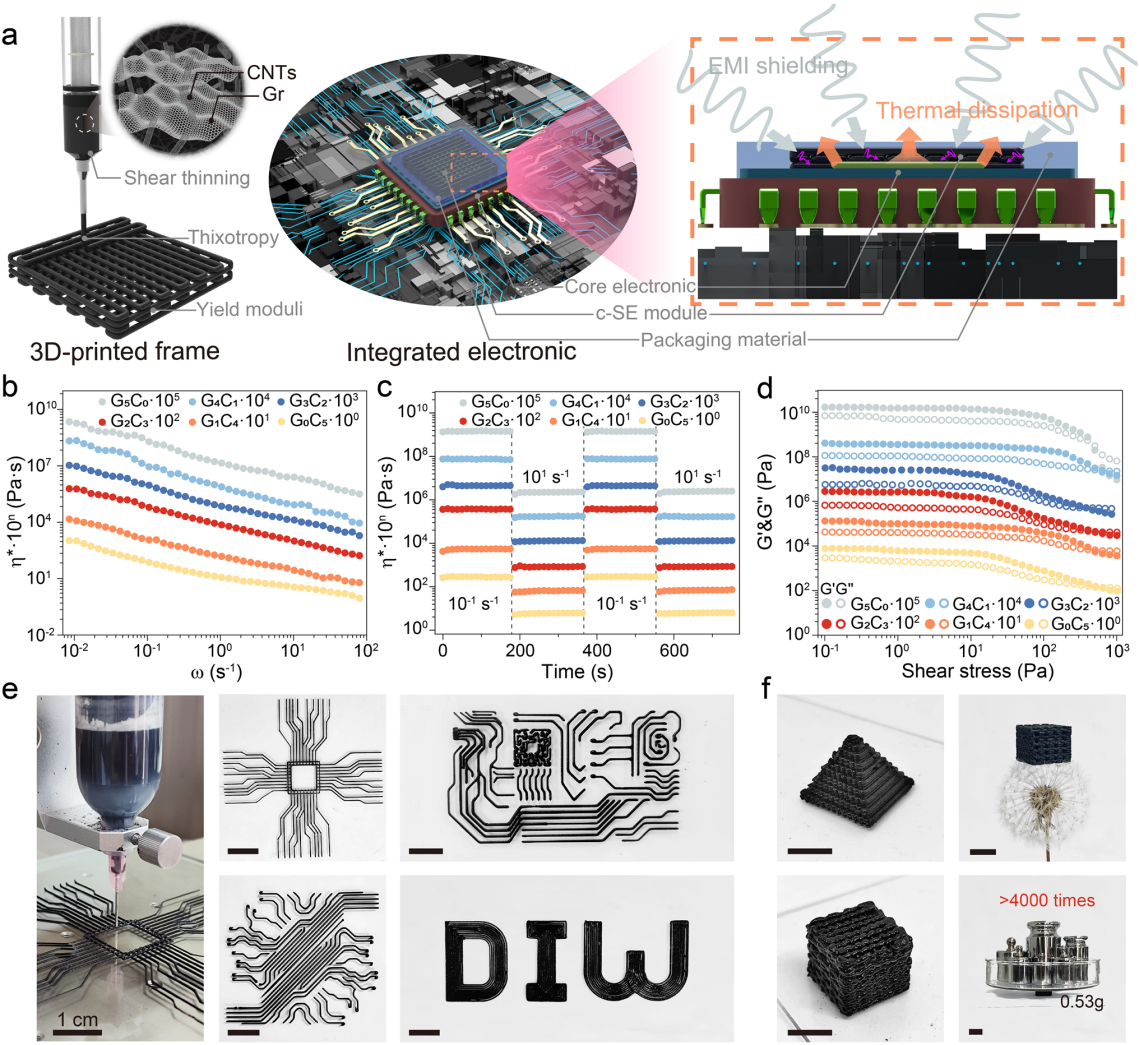
Rheological performance of Gr@CNT functional inks with various proportions. **a** Schematic of 3D printable Gr@CNT functional inks and their potential application in integrated electronics. **b** Complex viscosity (η*) as a function of oscillatory shear rate (*ω*). **c** η* upon the loads of two alternating shear rates of 0.01 and 10 s^−1^, respectively. **d** Moduli (*G*′ and *G*′′) as a function of oscillatory shear stress. **e, f** Digital images of 2D patterns and 3D architectures featuring ultralight and strong characteristics

A schematic regarding on the Gr@CNT functional inks and their potential application in integrated electronics is depicted in Fig. 1a, wherein Gr@CNT nanoparticles as functional mediums were well-formulated to uniformly disperse in CNF matrix for 3D printing. Noteworthily, as a characteristic biopolymer, CNF made a non-negligible contribution to the ink formulation, playing the multiple roles in adherent template, dispersing accelerant, and viscosity management. Due to the promising CH–*π* interaction between CNF and Gr@CNT nanoparticles [49], CNF molecules provided sufficient active sites for adsorbing nanoparticles to form the robust intertwined networks (Fig. S1). Besides, a negative surface charge owing to the carboxylate functional groups evidenced by the high zeta potential of CNF dispersion, exerted a strong electrostatic repulsion in molecular chains, which in turn improved the dispersion stability of Gr@CNT nanoparticles in CNF dispersion (Fig. S2). Moreover, the thickening effect of CNF molecules endowed the ink with appropriate viscoelastic characteristics for 3D printing. The pseudo-solid ink was strong enough to withstand its loading at a static state (Fig. S3), and meanwhile the smooth extrusion was realized through the narrow printing nozzle upon the suitable pressure (Fig. S4). Followingly, the rheological peculiarity of formulated inks with various Gr@CNT proportions are assessed in Fig. 1b–d. Generally, all inks performed ideal viscoelastic characteristics, including shear thinning, thixotropy and appropriate yield moduli. As shown in Fig. 1b, a typically shear-thinning behavior was demonstrated, suggesting a non-Newtonian peculiarity for the well-formulated inks, which is essential to uniformly flow out of a convergent nozzle under external pressure [50–52]. The power-law property is revealed synchronously in Fig. S5. The result further confirmed the viscoelastic characteristics of inks. Moreover, the thixotropic capability was investigated by examining the change in viscosity upon the alternating loads with a low (0.01 s^−1^) or high (10 s^−1^) shear rate (Fig. 1c). Each ink afforded a similar behavior, exhibiting a steady-state viscous contribution as fixed shear rate. Initially, a high viscosity over 10^2^ Pa s was detected in an extremely low shear rate (0.01 s^−1^) to simulate the pre-loading process, whereas the viscosity dropped below 10^1^ Pa·s when the shear rate increased to 10 s^−1^, indicating that the formed CNF and Gr@CNT intertwined networks were partially destroyed to resist the applied shear rate, thereby exhibiting a shear-thinning behavior. Meanwhile, the viscosity would rapidly recover to its initial state as the shear rate returned to the low one, and a perfect cycle was confirmed in response to the repeated shear rates. This reversible transformation in viscoelastic performance induced by shear rates provides a guarantee for inks with fluid appearance suitable for 3D printing and shape stability of 3D-printed architectures during depositing onto substrate [51]. Besides, the storage and loss moduli (*G*′ and *G*′′) of inks were examined by dynamic rheological scanning as a function of wide oscillatory stress (10^–1^ ~ 10^3^ Pa) in Fig. 1d. Generally, the moduli of all inks showed a similar tendency and presented a terminal plateau at the region below the yield point (*G*′ = *G*′′), implying that the networks were strong enough to withstand the loaded stress, performing a quasi-solid peculiarity. When the stress monotonously increased, the pseudo-solid inks were completely yielded and exhibited fluid characteristic, implying the partial broken of networks. Notedly, the appropriate yield moduli would endow the inks with ideal 3D printability and robust supportability, thus laying an essential foundation for the layer-by-layer printing [52]. Thus, these ideal rheological behaviors fully demonstrate that the 3D printability of Gr@CNT functional inks with the assistance of CNF.

Thereafter, the representative ink with Gr@CNT proportion of 2:3 (signed as G_2_C_3_) was employed to experimentally evaluate 3D printability. According to the systematic trajectories, the various 2D patterns were designed and printed in Fig. 1e. The well-organized shapes demonstrated that the ink possessed desired printability for manufacturing arbitrarily-customized geometries. Moreover, the 3D architectures including pyramid and lattice structures were programmatically assembled by layer-by-layer stacking (Fig. 1f). In addition, due to the extremely low density of Gr@CNT nanoparticles and CNF, the assembled architecture imparted ultralight characteristic (~ 0.076 g cm^−3^), and simultaneously afforded more than 4000 times its own weight without any destruction and collapse. Besides, with the same loading, the good shape-stability of 3D architecture was demonstrated under a relatively high-temperature environment to simulate the effects of the internal heating of electronics on designed structure (Fig. S6). Hence, these physical characteristics provide essential guarantees for the fabrication of high-performance functional frames via 3D printing technique.

### Structural Characterizations of 3D-Printed Frames

**Fig. 2:**
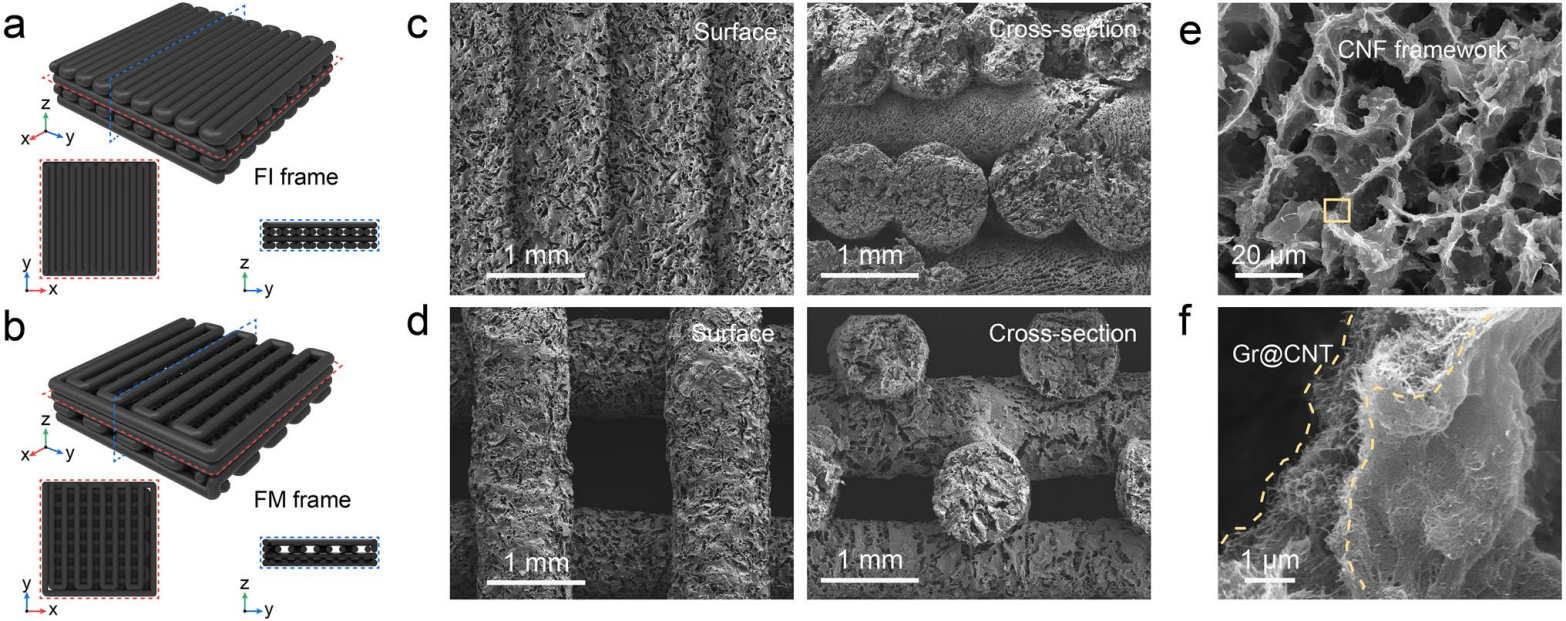
Morphologies of 3D-printed full-filling (FI) and full-mismatch (FM) frames with G_2_C_3_ ink. **a, b** Schematic of 3D printing FI and FM models. **c, d** SEM images of the surface (left) and cross-section (right) structures. **e, f** The high-resolution SEM images of FM sample

Owing to the intrinsic electrical performance of Gr and CNT nanoparticles, the assembled architectures are anticipated to exploit their potentialities in electromagnetic compatibility for integrated electronics. In this context, the representative 3D-printed frames with various stacking modes signed as full-filling (FI) and full-mismatch (FM) architectures are depicted in Fig. 2a, b. Accordingly, the surface and cross-section morphologies of as-designed frames are exhibited in Fig. 2c, d, and the regular stacks of FI and FM structures manifested the well tailorability of free-constructed shapes via 3D printing, whether in full-filling or full-mismatch modes. Moreover, the artificial porous structures were densely distributed in the interior of frames, exhibiting the lightweight characteristic for frame. In addition, the high-resolution SEM images in the cross-section of FM architecture are performed in Fig. 2e, f. As displayed in Fig. 2e, the porous structures assembled by CNF were formed and provided sufficient sites for the loading of CNT and Gr nanoparticles. More visualized in Fig. 2f, a large number of Gr@CNT nanoparticles were densely entangled on the CNF frameworks, which is mainly attributed to the interaction between CNF and Gr@CNT nanoparticles [27, 49].

### EMI SE peculiarity of 3D-Printed Frames

**Fig. 3:**
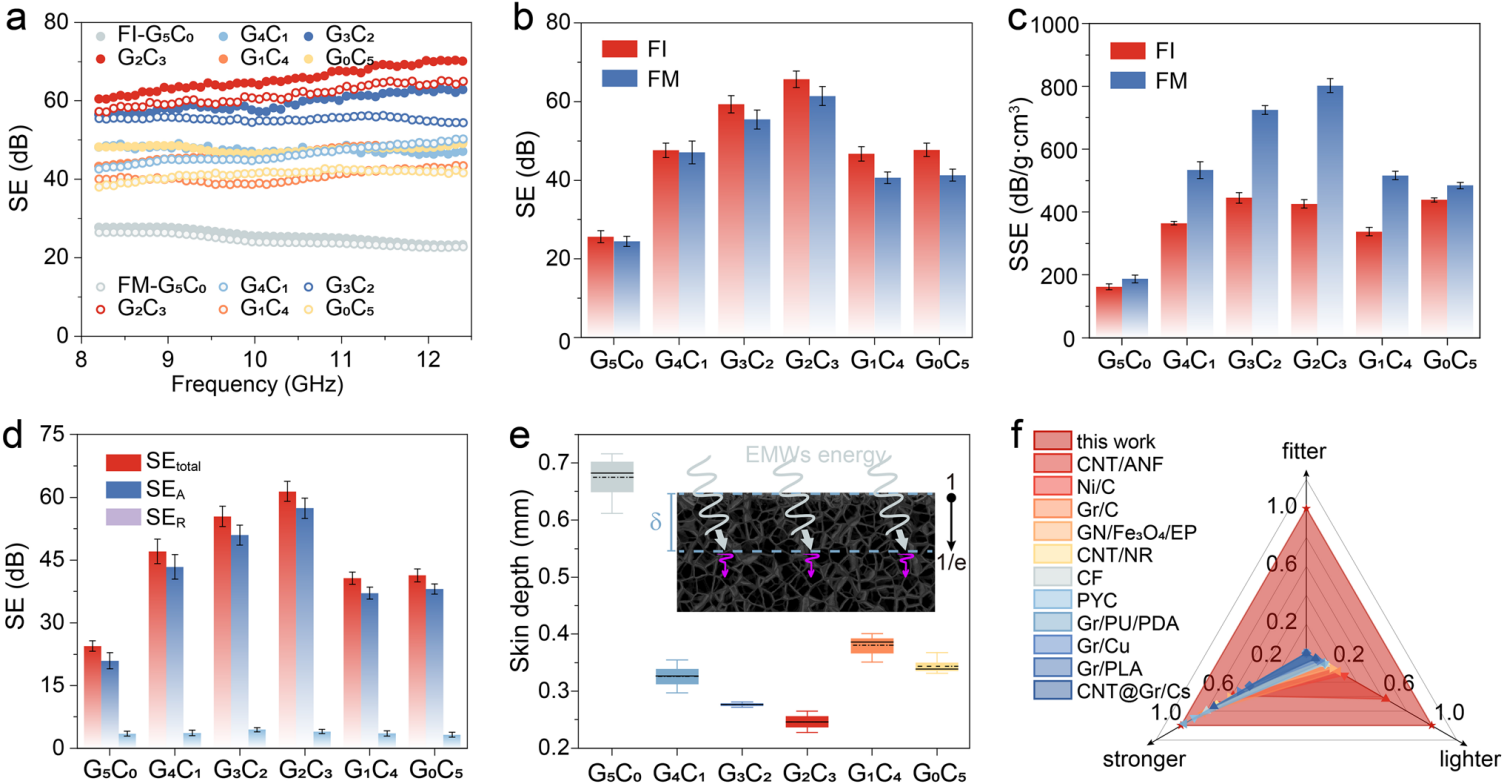
EMI SE peculiarity of 3D-printed FI and FM frames with various Gr@CNT proportions. **a** EMI SE properties in the X-band frequency range. **b, c** The average EMI SE and SSE values. **d, e** The electromagnetic parameters (SE_total_, SE_A_, and SE_R_) and skin depth (*δ*) of FM frames. **f** A radar plot benchmarking the key parameters (lighter, stronger, and fitter features) of 3D-printed FM frame in this work and other SE materials previously reported in the literatures (the references inside this plot listed in Table S3, ANF: aramid nanofiber, PU: Polyurethane, Cs: Carbon-matrix nanocomposites, CF: carbon foam, NR: Natural rubber, PLA: Polylactic acid, PYC: Pyrolytic carbon, GN: Graphene nanosheets, EP: Epoxy). (The inset in **e** shows schematic of energy dissipation of EMWs to *e*^−1^)

Generally, the intertwined networks of Gr@CNT nanoparticles would endow the materials with robust electrical conductivity, which plays a positive contribution on EMI SE performance [28, 53, 54]. The SE behaviors of 3D-printed frames with various stacking modes assembled by the as-formulated functional inks are evaluated in Fig. 3. As displayed in Fig. 3a, a similar tendency for shielding properties of 3D-printed frames was revealed, whether in FI or FM modes, i.e., the EMI SE value climbed first and then decreased with the sequential replacement of two-dimensional Gr by one-dimensional CNT nanoparticles. Typically, the optimum one of FI-G_2_C_3_ sample was higher than 60 dB in whole X-band region, corresponding to an extremely low transmittance of 0.000001 for EMWs energy, which far exceeds commercial SE standard (20 dB) [31, 55]. This outstanding SE behavior was mainly attributed to the excellent electrical performance of intertwined networks assembled by Gr@CNT nanoparticles (Fig. S7). To make a visualized comparison, the average EMI SE and SSE of all FI and FM frames are calculated in Fig. 3b, c. Generally, the EMI SE value of FI sample was slightly better than that of FM sample at the same Gr@CNT proportion, which is predominantly attributed to a richer number of pores, imparting longer propagation paths to EMWs, thereby affording a higher internal dissipation of EMWs energy [56]. Nevertheless, the ignorable sacrifice for the EMI SE value of FM samples was in exchange for a significant enhancement in SSE performance. For instance, the SSE value of FM-G_2_C_3_ frame was up to 802.4 dB cm^3^ g^−1^, 88.4% higher than that of FI-G_2_C_3_ one, meanwhile the total SE value of FM-G_2_C_3_ frame still maintained at 61.4 dB as compared with the FI-G_2_C_3_ one (65.7 dB). Particularly, for the conventionally compacted sample with the same Gr@CNT proportion and weight, the EMI SE and SSE values were only 44.2 dB and 63.9 dB cm^3^ g^−1^, respectively, implying the significant advantage of designed porous structures than solid structures (Fig. S8). Taken together, the 3D-printed FM frames made an excellent reconciliation in lightweight structure and high-performance SE behavior, thus realizing lighter and stronger shielding characteristics for potential shielding application. Moreover, owing to the free construction via 3D printing, the designed frames also possessed fitting feature for assembling arbitrarily-customized architectures onto integrated electronics.

Followingly, the potential shielding mechanism of 3D-printed frames was investigated by assessing the relevant shielding parameters including SE_total_, SE_A_, and SE_R_ (Figs. 3d and S9). Whether in FI or FM frames, SE_A_ followed the change of SE_total_ sequentially, while SE_R_ maintained a nearly constant value, below 5 dB, i.e., the characteristic of SE_A_ played a dominating role for the total shielding property (SE_total_) of as-designed carbon-based frames. The essence is mainly due to the positive contribution of the porous structures to the electromagnetic interference shielding performance of materials. As EMWs is transmitted into the shielding material, the porous structures allow EMWs to travel a longer distance inside the materials, thus promoting the SE_A_ by increasing the reflection and multiple reflections inside the pores, and attenuating the EMWs energy as Joule heat [53, 56]. More importantly, another parameter of skin depth (δ) representing for the depth where the EMWs energy dissipates to *e*^−1^ [43] is further evaluated in Fig. 3e. Obviously, all FM frames existed an extremely low *δ*, and the optimum one was down to ~ 246 μm, implying that the micron-level thickness of FM frame could be responsible for SE requirements, well-fitting for the application in integrated electronics. More importantly, the trinity advantages of 3D-printed frames on “lighter-stronger-fitter” shielding features were demonstrated as compared to other EMI SE materials previously reported in the literature (Fig. 3f) (the detailed references inside this plot are listed in Table S3).

### Electrothermal Performance of 3D-Printed Frames

**Fig. 4:**
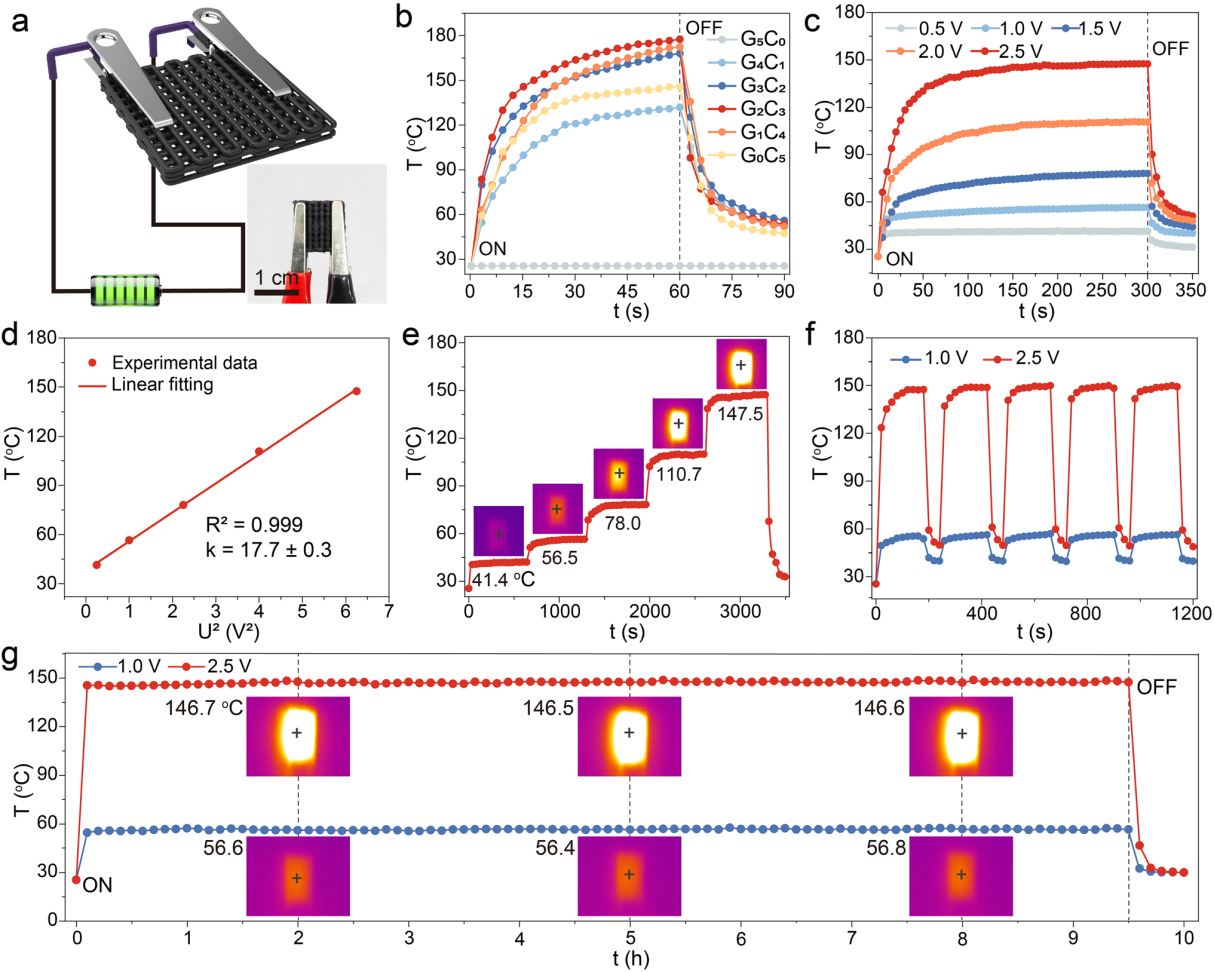
Electrothermal performance of 3D-printed FM frames with various Gr@CNT proportions. **a** Schematic of electrothermal energy conversion. **b** Joule heating performance at a fixed voltage of 3 V. **c, d** Joule heating performance of FM-G_2_C_3_ frame upon multiple input voltages from 0.5 to 2.5 V, and the corresponding linear fit of equilibrium temperature vs. square of input voltage (*U*^2^). **e** The real-time temperature of FM-G_2_C_3_ frame by loading the step-wise voltages from 0.5 to 2.5 V. **f** The temperature of FM-G_2_C_3_ frame upon the multiple cycling tests at two fixed voltages of 1.0 and 2.5 V. **g** The long-time electrothermal stability of FM-G_2_C_3_ frame at two fixed voltages of 1.0 and 2.5 V over 10 h. (the insets in **e** & **g** shows the representative infrared images)

The porous structures of 3D-printed frames as well as the densely intertwined Gr@CNT nanoparticles provided a promising potential in the thermal management of electronics under complicated thermal environments, e.g., high-efficiency heat compensation/dissipation capabilities [29, 44, 57, 58]. Hence, a schematic concerning on the evaluation of electrothermal performance of 3D-printed FM frames is depicted in Fig. 4a. Initially, the Joule heating performance of FM frames with various Gr@CNT proportions was investigated under a fixed voltage of 3 V (Fig. 4b). Apart from FM-G_5_C_0_ frame, others presented a similar tendency, that the time-dependent temperature increased first and then reached an equilibrium, and the temperature was down to the initial stage as unloaded voltage. Comparatively, the FM-G_2_C_3_ frame possessed the optimal electrothermal behaviors, including high equilibrium temperature and remarkable heating/dissipating efficiencies. Thereafter, the multiple input voltages from 0.5 to 2.5 V were separately conducted on the FM-G_2_C_3_ sample in Fig. 4c. The superior electrothermal response time of less than 20 s was detected in the loading/unloading voltage process, suggesting the high heating/dissipating efficiencies. In addition, the differential equilibrium temperature was clearly observed, corresponding to 41.4, 56.5, 78.0, 110.6, and 147.4 °C, respectively. This strong voltage dependence was ascribed to the positive correlation between the equilibrium temperature and the square of input voltage (*U*^2^) in potential electrothermal energy conversion. Moreover, the linear correlation between *U*^2^ and temperature is revealed in Fig. 4d, which was accompanied by a coefficient of determination (*R*^2^) of 0.999, suggesting the stable impedance of frame during electrothermal measurement, thus providing a fundamental guarantee for their long-term thermal management application [59]. Thereafter, the generated temperature was detected in real time by loading step-wise voltages from 0.5 to 2.5 V in Fig. 4e, and the inset presented the representative infrared images. The step-wise and rapid response of temperature to various voltages laid an essential foundation on the tailorability of the electrothermal capability of 3D-printed frames, thereby using in the thermal management of electronics. The electrothermal reliability and stability are synchronously investigated in Fig. 4f, g. As shown in Fig. 4f, the multiple cycling measurements were conducted at two fixed voltages of 1.0 and 2.5 V, respectively. The steady step-change of temperature suggested the well repeatability for the thermal management of 3D-printed frame. What’s more, the long-time electrothermal stability of designed frame was confirmed by loading the same voltages over 10 h (Fig. 4g). Wherever inputting the voltages of 1.0 or 2.5 V, the equilibrium temperature almost maintained a constant, corresponding to about 56.6 and 146.6 °C, respectively, indicating the good thermal-stability of 3D-printed frame during the electrothermal measurement and excellent reliability for thermal management application. Hence, these ideal electrothermal behaviors posed the promising potentials in efficient heat compensation/dissipation capabilities for better serving electronics in complicated thermal thermal management environments.

### 3D-printed Conformal-shielding Module for Integrated Electronics

**Fig. 5:**
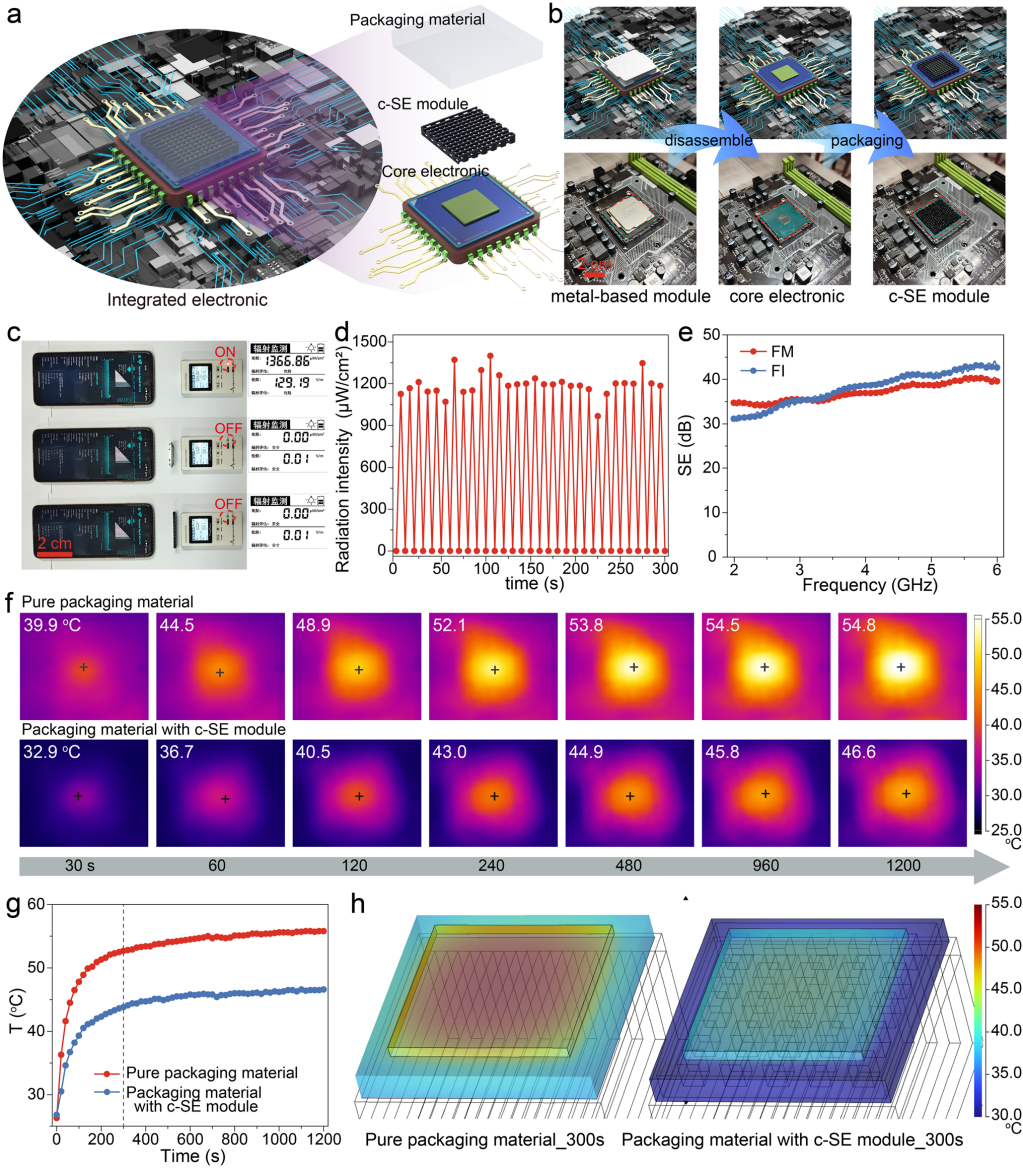
3D-printed c-SE module for integrated electronics. **a** Schematic of 3D-printed c-SE module onto core electronics. **b** The representative models and digital images including traditional metal-based module, disassembled core electronic, and 3D-printed c-SE module. **c** Digital images of the radiation intensity of EMWs signals before and after loading metal-based module or c-SE module. **d** Multiple cycles of the radiation intensity before and after loading c-SE module. **e** EMI SE performance of c-SE module in the 2.0–6.0 GHz frequency range. **f, g** The representative infrared thermal images of pure packaging material and the packaging material with c-SE module for assisting the thermal dissipation of electronics, and the corresponding real-time temperature curves. **h** COMSOL simulation of the thermal dissipation behaviors of pure packaging material and the packaging material with c-SE module

The 3D-printed frames have been fully demonstrated the prominent functionalities on EMI SE and thermal management capabilities. Toward the potential application of high-efficiency EMI shielding in integrated electronics, a novel alternative strategy regarding on 3D-printed conformal-shielding (c-SE) module was purposed, aiming to replace the traditional metal-based SE module. As a proof-of-concept, a schematic containing 3D-printed c-SE module with FM frame in-situ integrating onto core electronics by the assistance of packaging material is depicted in Fig. 5a. Accordingly, the representative models and digital images including traditional metal-based module, disassembled core electronic, and 3D-printed c-SE module are displayed in Fig. 5b. Owing to the outstanding capability of 3D printing technology in the customization of structure, the integrated c-SE module could be assembled the arbitrary-designated architecture to satisfy the demanding geometries for electronics. In addition, the good coordination between c-SE module and core electronic didn’t occupy the additional space to realize a positive contribution to the thermal management of the entire framework. Followingly, the principled capabilities of EMI shielding and thermal dissipation of assembled c-SE module are assessed in Fig. 5c–h. Notedly, due to operational difficulty in real-time monitoring EMWs shielding and thermal dissipation behaviors, the equivalent confirmatory experiments were designed to evaluate the functionalities of c-SE module. As displayed in Fig. 5c, a shielding detection regarding on the blocking of EMWs signal generated by electronics in mobile phone was carried out by a radiation tester. As anticipated, the designed c-SE module possessed the equivalent SE capability to traditional metal-based module disassembled from integrated electronics, exhibiting a completely shielding action to EMWs signal, where the corresponding radiation intensity was down from 1366.26 to 0 μT cm^−2^ as loaded c-SE module. Meanwhile, the multiple cycles were conducted to detect the radiation intensity before and after loading module in Fig. 5d. The pulse-like fluctuation of signal intensity indicated the well reliability of c-SE module for shielding EMWs inside electronics. Moreover, its SE performance in 2.0 ~ 6.0 GHz covering the commercial EMWs signals is recorded in Fig. 5e. The total shielding values were higher than 30 dB, fully satisfying the SE application for the commercial SE standard (20 dB) of electronics [44, 60]. Thereafter, the thermal dissipation contribution of c-SE module assembling with packaging material was equivalently evaluated on a LED heater, and the representative infrared images is exhibited in Fig. 5f (the measured digital image is shown in Fig. S10). As comparison to the pure packaging materials, commonly used in electronics for facilitating thermal dissipation, the packaging materials incorporated with c-SE module posed the better thermal dissipation efficiency than the pure one, and a maximal working-temperature difference could reach ~ 9 °C. Besides, the real-time temperature curves are synchronously provided in Fig. 5g. Indeed, the packaging materials with the assistance of c-SE module possessed a well thermal dissipation efficiency in the initial stage and a lower thermal equilibrium after reaching the energy balance, which is mainly attributed to the existing Gr@CNT networks inside packaging materials, leading to a high-efficiency thermal conduction for materials [29, 44, 58]. Moreover, the simulated results concerning on the thermal dissipation behavior in the same pre-set environment are investigated in Fig. 5h, and the detailed thermal-conductivity parameters and the geometrical grids are supported in Table S4, Figs. S11 and S12. Obviously, the resultant profiles confirmed the better capability of the packaging materials with c-SE module in facilitating the thermal dissipation of core electronics, which was consistent with the experimental results. Overall, these 3D-printed c-SE modules with high-efficiency EMI SE performance and well thermal management capability illuminate the infinite possibilities for assembling the next-generation multifunctional modules suitable for integrated electronics.

## Conclusions

In this study, to better serve the electromagnetic compatibility of integrated electronics, a novel Gr@CNT conformal-shielding (c-SE) module with arbitrarily-customized architectures and prominent functionalities was assembled by taking full advantages of 3D printing. Initially, the systematic experiments involving that the well-formulated inks featuring appropriate rheological capabilities were elaborately manipulated by incorporating various proportions of Gr and CNT nanoparticles into cellulose matrix. The as-prepared functional inks possessed ideal viscoelastic characteristics, taking charge of 3D printability. Thus, a series of 2D patterns and 3D architectures were free-constructed based on the pre-programmed printing trajectories. Meanwhile, the as-fabricated 3D-printed frames afforded expected functionalities, showing outstanding EMI SE performance and superior thermal management capability. As a representative, the optimum frame assembled by G_2_C_3_ ink exhibited an ultralight architecture (0.076 g cm^−3^) and remarkable SE capability (61.4 dB), as well as superhigh SSE peculiarity (802.4 dB cm^3^ g^−1^), far exceeding the reported carbon-based SE materials. Besides, the well tailorability for equilibrium temperature of designed frames was confirmed, imparting efficiency heat compensation/dissipation capabilities to electronics. What’s more, in order to expand the promising application of these high-performance architectures, an innovative concept concerning on 3D-printed c-SE module was purposed to replace traditional metal-based module to afford multiple functions for advanced electronics. The resultant behaviors of c-SE module on EMI SE performance and thermal dissipation full demonstrated their potentials on integrated electronics. Thus, the outstanding features of 3D-printed c-SE modules illuminate the infinite possibilities for assembling the next generation of high-performance carbon-based SE materials for integrated electronic.

## Supplementary Information

Below is the link to the electronic supplementary material.

Supplementary file1 (PDF 651 KB)
