# Supplementary material for: 3D-Printed Carbon-Based Conformal Electromagnetic Interference Shielding Module for Integrated Electronics
Source: Nanomicro Lett. 2024 Jan 12;16:85. doi: 10.1007/s40820-023-01317-w (PMC10786807; doi:10.1007/s40820-023-01317-w)
Supplement: Supplementary file 1 — Supplementary file1 (PDF 651 KB) [file 40820_2023_1317_MOESM1_ESM.pdf]

Supporting Information for

3D-Printed Carbon-Based Conformal Electromagnetic Interference Shielding Module for Integrated Electronics

Shaohong Shi[1, 2], Yuheng Jiang[1], Hao Ren[1], Siwen Deng[1], Jianping Sun[1], Fangchao Cheng[1, *], Jingjing Jing[2], Yinghong Chen[2, *]

[1] State Key Laboratory of Featured Metal Materials and Life-cycle Safety for Composite Structures, School of Resources, Environment and Materials, No. 100, Daxuedong Road, Guangxi University, Nanning 530004, P. R. China

[2] State Key Laboratory of Polymer Materials Engineering, Polymer Research Institute of Sichuan University, No. 24 South Section 1, Yihuan Road, Sichuan University, Chengdu 610065, P. R. China

*Corresponding authors. E-mail: fangchaocheng@gxu.edu.cn (Fangchao Cheng), johnchen@scu.edu.cn (Yinghong Chen)

Supplementary Figures and Tables

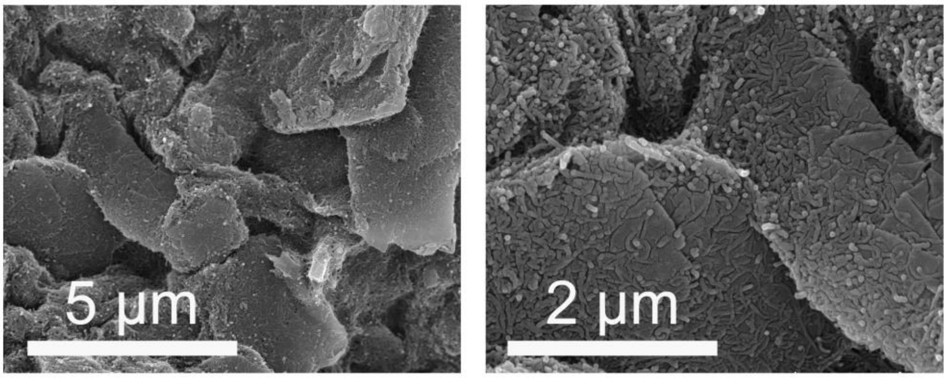

Fig. S1 SEM images of Gr@CNT intertwined networks in CNF framework

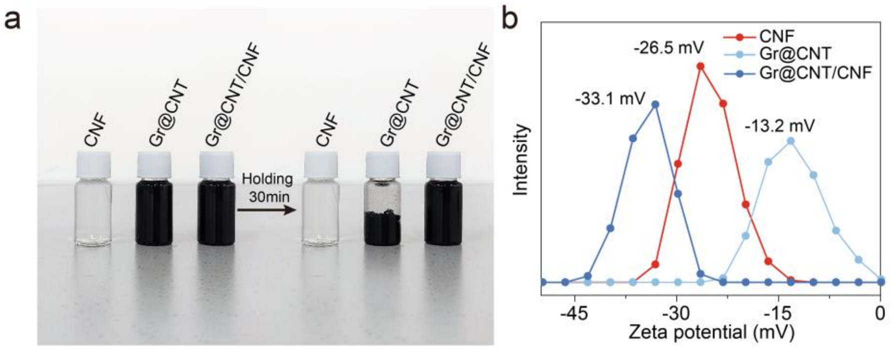

Fig. S2 a Digital image of the sedimentation experiment of CNF, Gr@CNT and Gr@CNT/CNF dispersions (~5.0 mg/mL) holding on 30 min, and **b** The corresponding Zeta potential of dispersions with a dilutional concentration of ~0.5 mg/mL

The sedimentation experiment of CNF, Gr@CNT and Gr@CNT/CNF dispersions before and after holding on 30 mins were conducted in Fig. S2a. Comparatively, the CNF and Gr@CNT/CNF dispersions still maintained in a stable state after holding on 30 min, whereas the obvious phase segregation occurred in Gr@CNT dispersion with the same holding time. This phenomenon implied that CNF molecules played a positive contribution on the dispersion of Gr@CNT nanoparticles in aqueous solution. Moreover, the corresponding Zeta potentials of these dispersions with a dilutional concentration of 0.5 mg/mL were measured by a Malvern Zetasizer NANO-ZS (Malvern Instruments, Worcestershire, U.K.) (Fig. S2b). The medium level of dispersion stability of CNF and Gr@CNT/CNF dispersions was verified, where the absolute Zeta potential was higher than 25 mV [S1].

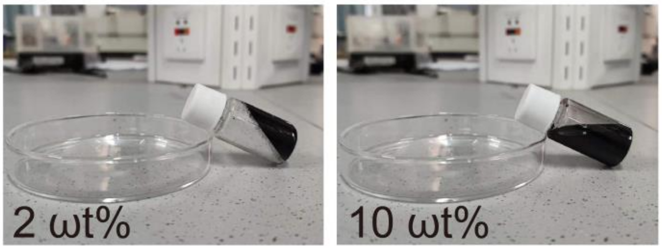

Fig. S3 Digital images of the inks with different solid contents

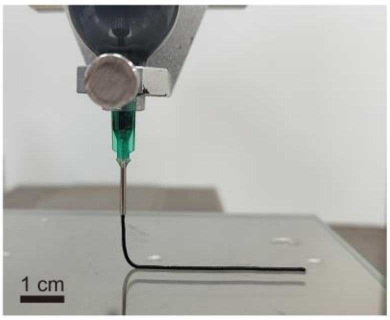

Fig. S4 Digital image of the smooth extrusion of Gr@CNT functional ink

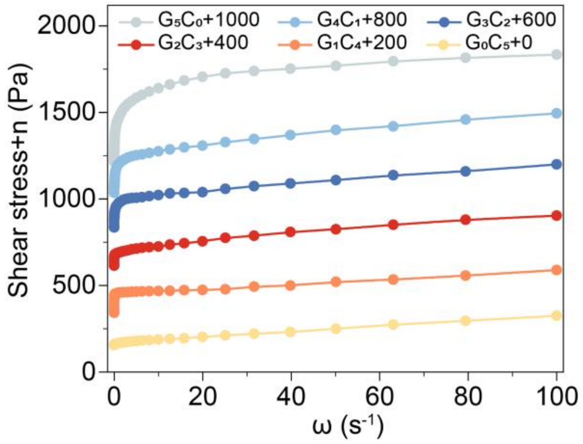

Fig. S5 Shear stress of the functional inks with various Gr@CNT proportions as a function of shear rate

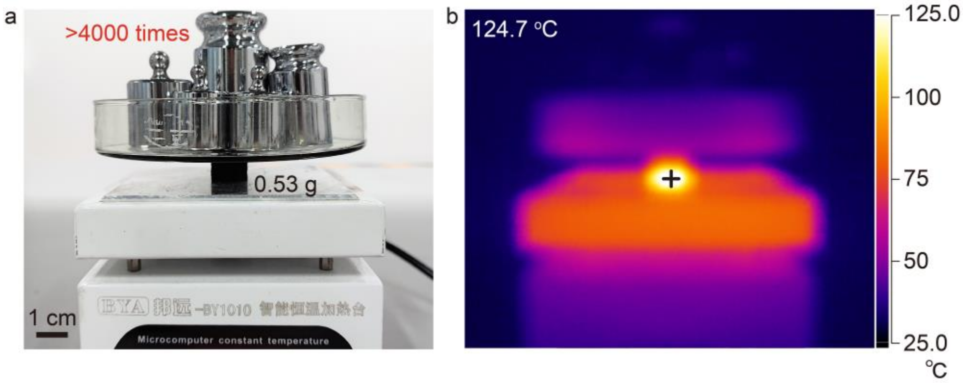

Fig. S6 Digital image and infrared image of 3D-printed frame under a relatively high-temperature environment

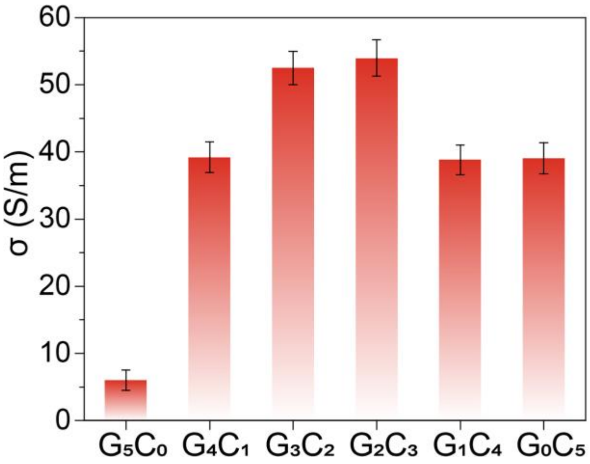

Fig. S7 The electrical conductivity of 3D-printed frames with various Gr@CNT proportions

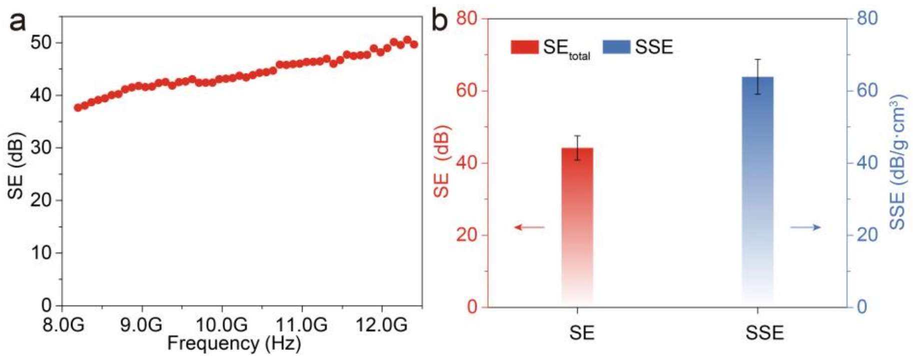

Fig. S8 a EMI SE property of the conventionally compacted sample in the X-band frequency range, and **b** The corresponding average EMI SE and SSE values

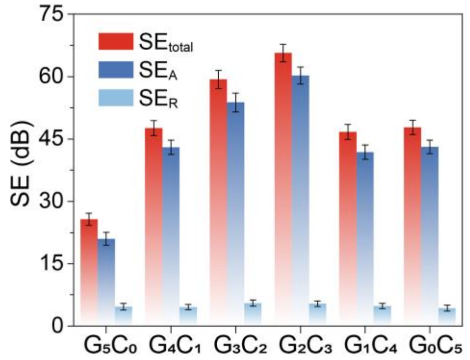

Fig. S9 The electromagnetic parameters (SE_{total}, SE_A, and SE_R) of 3D-printed FI frames

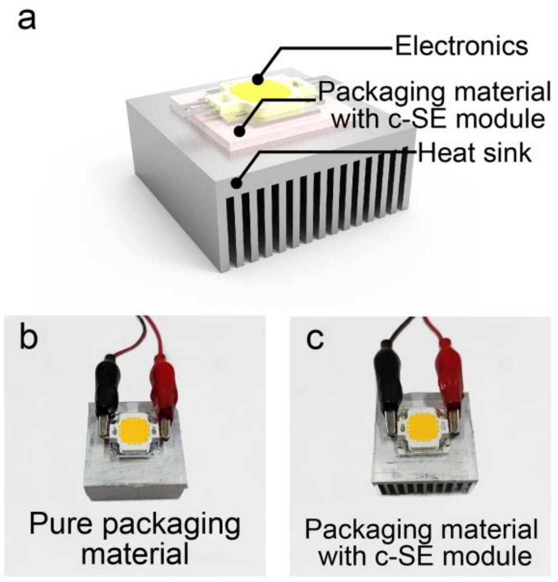

Fig. S10 a Schematic of the assembled thermal-dissipation models with electronic, packaging material with c-SE module, and heat sink. **b, c** Digital images of the tested samples with pure packaging material and packaging material with c-SE module

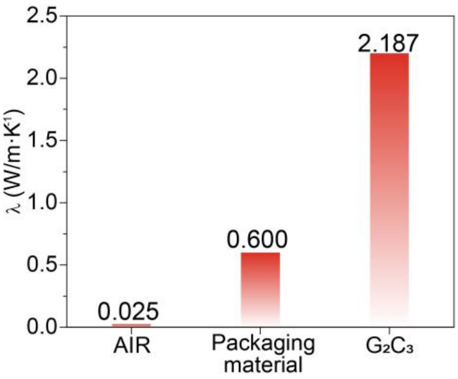

Fig. S11 The intrinsic thermal conductivity of air, packaging material and G_2C_3 sample

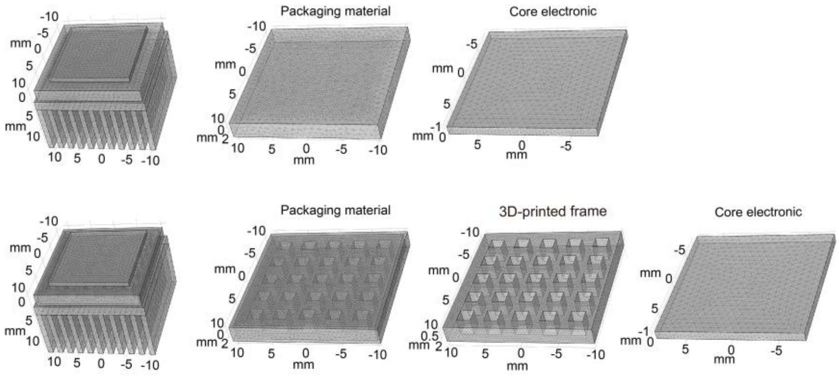

Fig. S12 Schematic of the meshes generated by the pure packaging material and the packaging material integrated with c-SE module

Table S1 The detailed compositions of carbon-based functional inks

Composition / Abbreviation	Gr / g	CNT / g	CNF / g	PVP / g
G_5C_0	1.0	0.0	0.3	0.15
G_4C_1	0.8	0.2	0.3	0.15
G_3C_2	0.6	0.4	0.3	0.15
G_2C_3	0.4	0.6	0.3	0.15
G_1C_4	0.2	0.8	0.3	0.15
G_0C_5	0.0	1.0	0.3	0.15

(Gr: graphene, CNT: carbon nanotube, CNF: cellulose, PVP: polyvinylpyrrolidone)

Table S2 The detailed printing information

X/Y Axis movement speed (mm/s)	Z Axis movement speed (mm/s)	X/Y axis movable limit (mm)	Z axis movable limit (mm)	Syringe volume (mL)
40	20	150	50	30

Table S3 Comparison of the lighter, stronger, and fitter characteristics of 3D-printed Gr@CNT EMI SE frame in this work and other SE materials previously reported in the literature

**Composites**	**ρ (g/cm^3)**	**EMI SE (dB)**	**Refs.**
Ni/C	0.25	43.1	[S2]
CNT/ANF	0.12	35.9	[S3]
Gr/PU	0.588	59.8	[S4]
Gr/Cu	0.72	32.6	[S5]
CNT@Gr/Cs	1.05	45.3	[S6]
Gr/C	0.30	50.7	[S7]
CF	0.50	48.9	[S8]
CNT/NR	0.50	44.0	[S9]
Gr/PLA	0.98	27.8	[S10]
PYC	0.48	54.8	[S11]
GN/Fe_3O_4/EP	0.34	37.3	[S12]
**Gr@CNT/CNF**	**0.076**	**61.4**	**This work**

(CNT: carbon nanotube, ANF: aramid nanofiber, Gr: graphene, PU: Polyurethane, PDA: Polydopamine, Cs: Carbon-matrix nanocomposites, CF: carbon foam, NR: Natural rubber, PLA: Polylactic acid, PYC: Pyrolytic carbon, GN: Graphene nanosheets, EP: Epoxy)

Table S4 The simulation parameters and boundary conditions (COMSOL Multi-physics)

Item	Heat source (Core electronic)	Heat sink material (Packaging material)	Heat sink material (3D printing frame)
Sample size (x×y×z, mm^3)	16×16×1	21×21×2.5	21×21×2.5
Thermal conductivity (W·m^{-1}·K^{-1})	1.38	0.60	2.187
Specific heat capacity (J·kg^{-1}·K^{-1})	703.00	1000.60	999.88
Surface emissivity (%)	0.80		
Heat power (W)	1.00	/	
Initial temperature (K)	293.15		

Supplementary References

[S1]M. K. Chauhan, P. K. Sharma. Optimization and characterization of rivastigmine nanolipid carrier loaded transdermal patches for the treatment of dementia. Chem. Phys. Lipids **224**, 104794 (2019). https://doi.org/10.1016/j.chemphyslip.2019.104794

[S2]Y. Zheng, Y. Song, T. Gao, S. Yan, H. Hu et al., Lightweight and hydrophobic three-dimensional wood-derived anisotropic magnetic porous carbon for highly efficient electromagnetic interference shielding. ACS. Appl. Mater. Interfaces **12**(36), 40802-40814 (2020). https://doi.org/10.1021/acsami.0c11530

[S3]C. Fu, Z. Sheng, X. Zhang. Laminated structural engineering strategy toward carbon nanotube-based aerogel films. ACS Nano **16**(6), 9378-9388 (2022). https://doi.org/10.1021/acsnano.2c02193

[S4]D. Fan, N. Li, M. Li, S. Wang, S. Li et al., Polyurethane/polydopamine/graphene auxetic composite foam with high-efficient and tunable electromagnetic interference shielding performance. Chem. Eng. J. **427**, 131635 (2022). https://doi.org/10.1016/j.cej.2021.131635

[S5]A. Yan, Y. Liu, Z. Wu, X. Gan, F. Li, et al., RGO reinforced Cu foam with enhanced mechanical and electromagnetic shielding properties. J. Mater. Res. Technol. **21**, 2965-2975 (2022). https://doi.org/10.1016/j.jmrt.2022.10.119

[S6]L. Feng, Y. Zuo, X. He, X. Hou, Q. Fu et al., Development of light cellular carbon nanotube@graphene/carbon nanocomposites with effective mechanical and EMI shielding performance. Carbon **168**, 719-731 (2020). https://doi.org/10.1016/j.carbon.2020.07.032

[S7]P. R. Agrawal, R. Kumar, S. Teotia, S. Kumari, D. P. Mondal et al., Lightweight, high electrical and thermal conducting carbon-rGO composites foam for superior electromagnetic interference shielding. Compos. Part B-Eng. **160**, 131-139 (2019). https://doi.org/10.1016/j.compositesb.2018.10.033

[S8]V. K. Patle, Y. Mehta, N. Dwivedi, D. P. Mondal, A. K. Srivastava et al., Thermal insulating and fire-retardant lightweight carbon-slag composite foams towards absorption dominated electromagnetic interference shielding. Sustain. Mater. Techno. **33**, e00453 (2022). https://doi.org/10.1016/j.susmat.2022.e00453

[S9]Z. Xie, Y. Cai, Y. Zhan, Y. Meng, Y. Li et al., Thermal insulating rubber foams embedded with segregated carbon nanotube networks for electromagnetic shielding applications. Chem. Eng. J. **435**, 135118 (2022). https://doi.org/10.1016/j.cej.2022.135118

[S10] Z. Peng, Q. Lv, J. Jing, H. Pei, Y. Chen et al., FDM-3D printing LLDPE/BN@GNPs composites with double network structures for high-efficiency thermal conductivity and electromagnetic interference shielding. Compos. Part B-Eng. **251**, 110491 (2023). https://doi.org/10.1016/j.compositesb.2022.110491

[S11] X. Liu, H. Liu, H. Xu, W. Xie, M. Li et al., Natural wood templated hierarchically cellular NbC/pyrolytic carbon foams as stiff, lightweight and high-performance electromagnetic shielding materials. J. Colloid. Interf. Sci. **606**, 1543-1553 (2022). https://doi.org/10.1016/j.jcis.2021.08.110

[S12] H. Liu, C. Liang, J. Chen, Y. Huang, F. Cheng et al., Novel 3D network porous graphene nanoplatelets /Fe_3O_4/epoxy nanocomposites with enhanced electromagnetic interference shielding efficiency. Compos. Sci. Technol. **169**, 103-109 (2019). https://doi.org/10.1016/j.compscitech.2018.11.005
